# Retention Time Extended by Nanoparticles Improves the Eradication of Highly Antibiotic-Resistant *Helicobacter pylori*

**DOI:** 10.3390/pharmaceutics14102117

**Published:** 2022-10-05

**Authors:** Cheng-Jung Yao, Shu-Jyuan Yang, Chung-Huan Huang, Yuan-Ting Chang, Chung-Hao Wang, Ming-Jium Shieh, Tai-Horng Young

**Affiliations:** 1 Institute of Biomedical Engineering, College of Medicine and College of Engineering, National Taiwan University, No. 1, Section 1, Jen-Ai Road, Taipei 100, Taiwan; 2 Division of Gastroenterology, Department of Internal Medicine, Wan Fang Hospital, No. 111, Section 3, Xinglong Road, Taipei 116, Taiwan; 3 Gene’e Tech Co., Ltd., 2nd Floor, No. 661, Bannan Road, Zhonghe District, New Taipei City 235, Taiwan; 4 Department of Oncology, National Taiwan University Hospital and College of Medicine, No. 7, Chung-Shan South Road, Taipei 100, Taiwan

**Keywords:** *Helicobacter pylori*, amoxicillin, chitosan, alginate, nanoparticle

## Abstract

*Helicobacter pylori* infection usually causes gastrointestinal complications, including gastrointestinal bleeding or perforation, and serious infections may lead to gastric cancer. Amoxicillin is used to treat numerous bacterial infections but is easily decomposed in the gastric acid environment via the hydrolyzation of the β-lactam ring. In this study, we develop chitosan-based nanoparticles loaded with amoxicillin (CAANs) as an *H. pylori* eradication platform. The CAANs were biocompatible and could retain the antibiotic activity of amoxicillin against *H. pylori* growth. The mucoadhesive property of chitosan and alginate enabled the CAANs to adhere to the mucus layers and penetrate through these to release amoxicillin in the space between the layers and the gastric epithelium. The use of this nanoparticle could prolong the retention time and preserve the antibiotic activity of amoxicillin in the stomach and help enhance the eradication rate of *H. pylori* and reduce treatment time. These CAANs, therefore, show potential for the effective treatment of highly antibiotic-resistant *H. pylori* infection using amoxicillin.

## 1. Introduction

*Helicobacter pylori (H. pylori)* was discovered in 1983 by Marshall and Warren and is a spiral Gram-negative bacterium with cilia [1]. It is mainly transmitted via dirty water, food, and saliva. The prevalence rate of infection varies slightly with the degree of development in the country, with an infection rate of approximately 90% in developing countries [2]. After infection, *H. pylori* can secrete urease to break down urea into carbon dioxide and ammonia to provide protection against the damaging effect of gastric acid. The immune response against *H. pylori* infection can induce chronic inflammation that can damage the protective mechanism of the stomach and result in peptic ulcers. Without proper treatment, *H. pylori* infection may cause serious complications, such as gastrointestinal bleeding or perforation, and the most serious infections may cause more serious stomach problems, such as gastric cancer. Therefore, *H. pylori* is considered a Group 1A carcinogen by the International Agency for Research on Cancer [3].

Standard triple therapy is now widely used as the first-line regimen for the eradication treatment of *H. pylori*; it consists of proton pump inhibitors (PPI) and two first-line antibiotics, including clarithromycin, amoxicillin, and metronidazole. Standard triple therapy initially had an *H. pylori* eradication rate as high as 90%, but the current eradication success rate is lower than 80% in many countries in the face of the rising prevalence of antibiotic resistance [4]. To increase the eradication rate of *H. pylori* with increasing clarithromycin resistance, quadruple therapy, including the use of bismuth, may be preferable [5]. However, the use of the quadruple method is hindered by the complexity of the administration method and the higher incidence of side effects than those observed with triple therapy. Amoxicillin, a β-lactam penicillin antibiotic, is used to treat numerous bacterial infections [6,7]. Although the prevalence of *H. pylori* resistance is low for amoxicillin at 3%, the antibiotic activity is easily destroyed in the gastric acid environment by hydrolysis of the β-lactam ring [8,9,10]. High doses and/or more frequent PPI and amoxicillin administration are used to increase the antibiotic concentration in plasma against *H. pylori* [11]. However, high-dose triple therapy usually causes unpleasant symptoms that decrease patient compliance with the treatment [12].

**Scheme 1:**
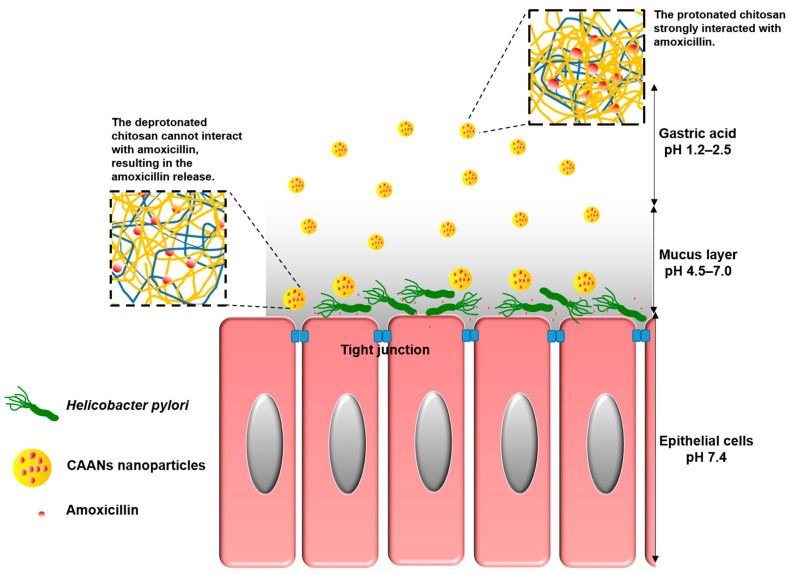
Schematic illustration showing that CAANs are stable in the gastric acid of the gastric lumen; they penetrate the mucus layer, become unstable, and then release the amoxicillin at the site of *H. pylori* infection.

To allow oral amoxicillin to directly act on *H. pylori* in the stomach and protect the amoxicillin against gastric acid-induced hydrolysis, several mucoadhesive polymers, such as chitosan, carboxymethylcellulose, or alginate, can be used to encapsulate amoxicillin in an amoxicillin delivery system [13]. Chitosan is a natural polymer consisting of 2-amino-2-deoxy-β-d-glucan by glycosidic linkages and can be obtained via the alkaline deacetylation of chitin [14]. Since chitosan can be dissolved in weakly acidic aqueous solutions, it can be widely applied in the form of films, beads, fibers, or gels, according to the intended use. Chitosan also exhibits excellent biodegradable properties, is nontoxic and nonallergenic, and contains a variety of reactive amine and hydroxyl groups that can be conjugated with other molecules for specific pharmaceutical applications, including drug delivery, wound dressing, artificial liver development, nerve repair, and antibacterial material development [15]. Moreover, the mucoadhesive property of chitosan makes it highly useful in applications of gastrointestinal, buccal, and nasal mucosal drug delivery systems [16,17,18,19,20,21,22]. Therefore, in this study, we encapsulate amoxicillin in chitosan-based nanoparticles (CAANs) that would protect the amoxicillin against hydrolysis by gastric acid; the nanoparticles would then attach to and pass through the mucus layer to release the loaded amoxicillin to act on *H. pylori* directly (Scheme 1).

Sodium alginate, also known as kelp gum or alginate, is a natural polyanionic copolymer of 1–4-linked β-d-mannuronic acid and α-l-glucuronic acid and can be used in beverage clarifiers and jam thickeners and as bread and noodle quality optimizers [23]. Due to favorable biocompatibility and biodegradability, low toxicity, and superior gelling formation with divalent cations, alginate is widely used in pharmaceutical medicine [24,25]. Moreover, alginate can be used as a solid support carrier to immobilize cells for applications in fermentation engineering [26]. Previously, we have incorporated alginate into 5-aminolaevulinic acid (5-ALA)-loaded chitosan nanoparticles to enhance the release of 5-ALA into the lysosome via competition for the chitosan cationic residues [26]. In this study, we expect that the incorporated alginate could not only enhance the retention time of the CAANs in the stomach by utilization of the mucoadhesive property but could also improve amoxicillin release when the prepared particles penetrate through the mucus layer to enable the improved eradication of *H. pylori* in both in vitro and in vivo models.

## 2. Materials and Methods

### 2.1. Materials

Chitosan with Mw of 15,000 Da (≥95%) was purchased from Polysciences, Inc. (Warrington, PA, USA). Alginic acid sodium salt from brown algae (low viscosity), amoxicillin (potency ≥ 900 μg/mg), omeprazole (≥98%), clarithromycin (≥95%), and mucin from porcine stomach were purchased from Sigma-Aldrich (St. Louis, MO, USA). Sodium tripolyphosphate (STPP, 95%) was acquired from Wako Pure Chemical Industries (Osaka, Japan).

### 2.2. Preparation of CANs and CAANs

The preparation of amoxicillin encapsulated in chitosan–alginate nanoparticles (CAANs) was based on the ionic gelation interaction of positively charged chitosan in the existence of negatively charged STPP and alginate [26]. At the same time, ionized amoxicillin with negative charges can also partially contribute to the gelation interaction with chitosan. First, 0.05% chitosan solution was readied by dissolving 0.5 mg chitosan powder in 1 mL of 0.01 M acetic acid solution at pH 4.0. A solution of 0.15% amoxicillin was prepared by dissolving 1.5 mg amoxicillin powder in 1 mL 0.005% alginate solution, in which alginate was pre-dissolved in a 0.01 M NaOH solution containing 0.05% STPP, with the pH adjusted to 7.4 with HCl. Alginate solution or amoxicillin/alginate solution (2 mL) was dropped into 5 mL of chitosan solution at a flow rate of 0.5 mL/min using a peristaltic pump to prepare CANs or CAANs, respectively. The prepared CANs and CAANs suspended in the solution were later used directly without post-treatment requirements. 

### 2.3. Characterization of CANs and CAANs

The average particle size and surface charge of the prepared CANs and CAANs were measured with a Zetasizer Nano-ZS90 instrument (Malvern Instruments, Worcestershire, UK). Dried 200 mesh carbon-coated copper grids with CAN or CAAN precipitation were observed under a transmission electron microscope (TEM; Hitachi H-7500, Tokyo, Japan) to determine the morphology of the prepared nanoparticles.

The loading efficiency (LE) of amoxicillin in the prepared CAANs was evaluated via high-performance liquid chromatography (HPLC, Waters e2696). Briefly, after the preparation of CAANs and centrifugation at 16,000 rpm for 10 min, the supernatant was passed through a 0.22 µm filter to remove the particles that could not be separated by centrifugation, and 10 µL of the filtered supernatant was injected into the HPLC system. The mobile phase consisted of 5% acetonitrile at a flow rate of 1 mL/min, and the separation of the injected supernatant was achieved on a Waters Symmetry-C18 reversed-phase column (XBridgeTM). Un-encapsulated amoxicillin was investigated using a UV detector (229 nm). The drug’s LE was calculated using the following equation:

LE (%) = (Weight of the adding amoxicillin − Weight of the unloaded amoxicillin)/Weight of the adding amoxicillin × 100%

### 2.4. In Vitro Drug Release

The drug-release profiles of amoxicillin from the CAANs were investigated via the dialysis-bag diffusion method [27]. Briefly, 40 mL of the prepared nanoparticle solution was concentrated fivefold using Amicon^®^ Ultra centrifugal filter units (Millipore, Billerica, MA, USA) to separate the unloaded amoxicillin; then, 2 mL of concentrated nanoparticle solution was loaded into a 3.5 kDa cutoff CelluSep dialysis membrane (Membrane Filtration Products, Seguin, TX, USA). The nanoparticle-loaded dialysis membrane was immersed in 48 mL of buffer with pH 2.5, 4.0, 6.0, or 7.0 for 5 h at 37 °C to simulate the pH value in different stomach environments. After shaking at 100 rpm for a period, 1 mL of the buffer was taken out, and the optical density (OD) of the released amoxicillin was recorded via HPLC.

To observe the relationship between the morphological changes of the CAANs with different pH environments, 200 mesh carbon-coated copper grids were immersed in CAAN solutions at pH 2.5, 4.0, 6.0, or 7.0. The dried copper grids with CAANs were examined under a TEM. At the same time, the average particle size and zeta potential of the prepared nanoparticles in different pH environments were also measured using a Zetasizer Nano-ZS90 instrument.

### 2.5. In Vitro Mucoadhesion and Mucopenetration Evaluation

The in vitro mucoadhesion of the prepared nanoparticles with commercial porcine mucin particles was evaluated [28]; 1 mg of porcine mucin particles was dispersed in 1 mL deionized water and then mixed with an appropriate volume of the prepared CN (chitosan nanoparticles without loaded amoxicillin and incorporated alginate), CAN, or CAAN solutions at pH 2.5 to mimic the stomach environment. The change in the surface charge of the mucin particles was determined by measuring the zeta potential as above.

An additional evaluation was conducted using a method proposed by Abruzzo et al. with some modifications [29]. The prepared CN, CAN, or CAAN suspensions were well mixed with 1 mg/mL mucin dispersion (pH 2.5) at a volume ratio of 1:1, incubated at 37 °C for 60 min, and then centrifuged at 10,000 rpm for 30 min. The remaining free mucin in the supernatant was detected using an ultraviolet–visible (UV–vis) spectrophotometer (Cary 50 Conc; Varian, Palo Alto, CA, USA) at 261 nm.

To evaluate the mucopenetration of the prepared CAANs, 50 (pH 7.4) and 20 mg/mL (pH 4.5) porcine stomach mucin solutions were coated in turn on Costar^®^ transwell inserts (Corning Inc., Kennebunk, ME, USA) at a thickness of 200 μm to simulate the gastric mucosal system. The prepared CAAN solution was added at the top of the mucin layers. After 2 h at 50 rpm, the mucin layers were frozen at −20 °C and dried via lyophilization. The distribution of CAANs in the gastric mucosal systems was observed under a scanning electron microscope (SEM; Hitachi H-7650, Tokyo, Japan).

### 2.6. In Vitro Bacterial Growth Suppression

Four *H. pylori* strains (NO. 125-54, NO. 125-57, NO. 127-3, and NO. 127-5) were obtained from Jyh-Chin Yang from the Department of Internal Medicine at National Taiwan University Hospital. The minimum inhibitory concentration (MIC) values of amoxicillin with NO. 125-54, NO. 125-57, NO. 127-3, and NO. 127-5 were ≥0.5, 0.25, 0.0625, and 0.0625 μg/mL, respectively. For the in vitro growth suppression test, *H. pylori* strains with different MIC values were dispersed in Brucella broth (Sigma-Aldrich, St. Louis, MO, USA) with 10% fetal bovine serum and then co-incubated with free amoxicillin, CANs, or CAANs. After 48 h, the OD of Brucella broth containing *H. pylori* was measured at 600 nm [27]. Sterile and inoculated culture media were used as negative and positive controls, respectively. The percentage inhibition of bacterial growth was calculated using the following equation:

% inhibition of bacterial growth = 100 − (OD sample/OD positive control) × 100%

### 2.7. In Vitro Cytotoxicity

A mouse embryo fibroblast cell line (NIH/3T3) obtained from ATCC (NO. CRL-1658) was used as a standard cell line for biocompatibility assessment of the prepared nanoparticles. NIH/3T3 fibroblast cells were cultured in Dulbecco’s modified Eagle medium (GIBCO; Grand Island, NY, USA) supplemented with 10% (*v*/*v*) fetal bovine serum, 1.5 g/L sodium bicarbonate, 4.5 g/L glucose, 4 mM L-glutamine, and 1% (*v*/*v*) Gibco^®^ antibiotic–antimycotic solution (Thermo Fisher Scientific, USA) at 37 °C in a 5% CO_2_ atmosphere. The culture medium was changed every three days until 80% of the culture vessel area was covered with cells.

NIH/3T3 fibroblast cells were seeded at 1 × 10^4^/wells into 48-well culture plates and cultured for 24 h. The culture medium was replaced with fresh medium containing amoxicillin, CANs, or CAANs at amoxicillin concentrations of 5, 10, 100, and 1000 μg/mL. Herein, the CAN and CAAN solutions were concentrated 15-fold (Amicon Ultra, Millipore, Burlington, MA, USA) to remove the unloaded amoxicillin beforehand. After treatment for 48 h, the MTT assay was used to determine the cytotoxicity effect [27].

### 2.8. Location of CAANs in the Gastrointestinal Tract

Pathogen-free BALB/c mice (4 weeks old) were purchased from the National Laboratory Animal Center (Taipei, Taiwan). All experimental mice received care on the basis of the guidelines outlined in the Guide for the Care and Use of Laboratory Animals (8th edition). The in vivo experiments were conducted using protocols approved by the National Taiwan University College of Medicine and College of Public Health Institutional Animal Care and Use Committee (Approved NO. 20190127).

For the single-photon emission computed tomography (SPECT) study, amoxicillin was first radiolabeled with ^123^iodine (^123^I, emitting 159 KeV photons) using an idoge-tube (Pierce Iodination Tubes, Thermo Fisher Scientific, Rackford, IL, USA) [30] and then loaded into the prepared CAANs (^123^I-CAANs). BALB/c mice were fed with the ^123^I-amoxicillin or ^123^I-CAANs, and then SPECT/CT images were obtained using a NanoSPECT/CT (Bioscan Inc., Poway, CA, USA) at 4 and 24 h post oral administration. The mice were euthanized 24 h post oral administration, and their gastrointestinal tracts were removed. SPECT/CT images of the gastrointestinal tracts were then obtained.

### 2.9. Single Dose Pharmacokinetic Studies

Male CD^®^(SD)IGS rats (300–350 g) were purchased from BioLASCO Taiwan Co., Ltd. (Taipei, Taiwan). The animals were divided into two equal groups (n = 4). One group received the amoxicillin suspension via oral gavage, whereas the other group received the CAAN suspension via oral gavage. Amoxicillin was administered at 33 mg/kg. Blood samples (0.3 mL) were collected from the tail veins into microfuge tubes containing anti-coagulant (lithium heparin) at pre-dose and 1, 2, 3, 4, 5, 6, 7, 8, and 24 h post-dose. Plasma was separated from the blood components via centrifugation at 1500× *g* at 4 °C for 15 min and then stored at −75 °C until analysis via HPLC.

### 2.10. In Vivo Eradication Efficacy of H. pylori by CAANs

The development of the *H. pylori*-infected mouse model was performed by feeding 4 × 10^7^ CFU *H. pylori* (NO. 126-6, with an MIC value of 0.016 μg/mL) in BHI broth to each BALB/c mouse twice a day for two consecutive days; then, the infection was allowed to develop for 2 weeks. All the infected mice were randomly divided into three groups (n = 10); two of the groups were orally administered with phosphate-buffered saline (PBS) and CAANs, with a dose of 33 mg/kg amoxicillin, respectively. Administration was performed once daily for 7 consecutive days. The third group was orally treated twice daily with standard triple therapy, a combined regimen of omeprazole, amoxicillin, and clarithromycin, for 14 consecutive days. At 24 h post last administration, the mice were euthanized, and the stomachs were collected. Half the stomach tissue was rinsed with PBS, and then 1 mL of the BHI broth was added and ground with the tissue to a homogenized mix using mortar and pestle. The ground stomach tissue mix was coated onto the select agar plate surface and cultured at 37 °C under a microaerophilic condition for 2 days. Bacterial colonies were harvested using a sterile cotton swab and resuspended in PBS. To confirm the status of *H. pylori*, the resuspended bacterial solutions were analyzed via PCR to determine the CagA-gene expression, as previously described [27,31]. In order to confirm the eradication efficacy of CAANs on an *H. pylori* strain with higher antibiotic resistance, the *H. pylori* strain with an MIC value of 0.5 μg/mL (NO. 125-54) was also used to infect BALB/c mice, and then we simultaneously performed the above experiments to determine the eradication efficacy.

### 2.11. Statistical Analysis

Mean ± standard deviation was used to describe the data. Student’s *t*-test was employed to determine statistically significant differences between the groups. The results were considered significantly different at a *p*-value of <0.05.

## 3. Results and Discussion

### 3.1. Characterization of CANs and CAANs

**Figure 1:**
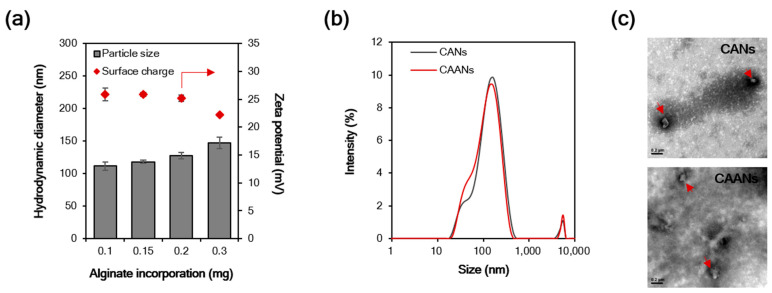
(**a**) Hydrodynamic diameter and surface charge of chitosan–alginate nanoparticles (CANs) with different alginate contents. (**b**) The size distribution of CANs and CAANs. (**c**) Transmission electron microscope images of CANs and CAANs (red arrow). Samples were negatively stained with 2% uranyl acetate before imaging. Scale bar = 200 nm.

**Figure 2:**
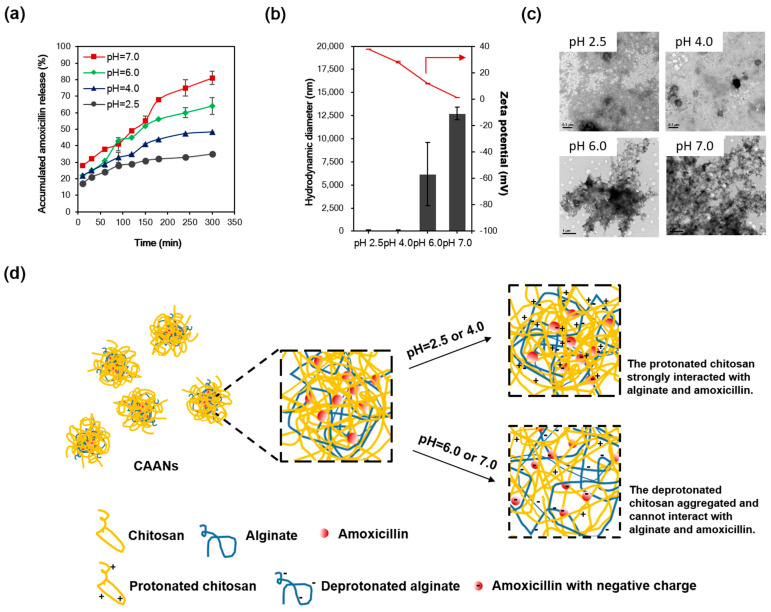
(**a**) The percent release profiles of the prepared CAANs at pH 2.5, 4.0, 6.0, and 7.0 and 37 °C. (**b**) Hydrodynamic diameter and zeta potential and (**c**) TEM images of the prepared CAANs at pH 2.5, 4.0, 6.0, and 7.0. (**d**) Schematic illustration of the amoxicillin release from the CAANs at pH 2.5, 4.0, 6.0, and 7.0 by decreasing the interaction of deprotonated chitosan with amoxicillin.

Particle size is an important influencing factor for the diffusion of particles in the gastric mucus layer due to the steric hindrance [32], and particles with a size larger than 200 nm cannot easily diffuse through the gastric mucosa [33]. In this study, the particle size of the prepared CANs positively correlated with the incorporated alginate content, although there was a reciprocal decrease in the zeta potential with the alginate content (Figure 1a). At 0.1 mg incorporated alginate, the CANs were approximately 111.7 nm in diameter, with a positive potential of 25.9 mV. When amoxicillin was loaded in the CAANs, no difference was observed in the particle size distribution (Figure 1b), but the zeta potential slightly decreased to 24.4 mV. Furthermore, the LE of amoxicillin in CAANs was 71.6%. Direct observation of the morphological features of CANs and CAANs using TEM (Figure 2c) revealed that the nanoparticles were not round but had a smooth surface. These results reveal that these designed CAANs, at sub-200 nm in diameter, may satisfy the steric requirement for rapid diffusion in and through the gastric mucus layer.

### 3.2. In Vitro Drug Release

Since amoxicillin is degraded most rapidly at the acidic pH of the stomach lumen [34], the combination of a higher dose of amoxicillin and PPI can be used to avoid antibiotic degradation in triple or quadruple therapy to increase the eradication rate of *H. pylori*. Here, the amoxicillin was protected in the prepared CAANs from degradation by gastric acid and was released in or under the mucus layer to kill or inhibit the growth of *H. pylori*. To investigate the amoxicillin release from the CAANs in the stomach, a drug-release test was conducted at pH 2.5, 4.0, 6.0, or 7.0 for 5 h at 37 °C to simulate the pH value in the gastric acid, outer and inner mucus layers, and gastric epithelium environment, respectively. As shown in Figure 2a, the release of amoxicillin from CAANs was dependent on the environmental pH. At a low pH (2.5 and 4.0), there was only a 30–40% release of amoxicillin from the CAAN, which may correspond to the non-encapsulated amoxicillin or amoxicillin that is only trapped in the particle surface, which is easy to diffuse from the CAANs due to the concentration difference. When the pH environment was higher than the pKa of chitosan (approximately 6.5), the amoxicillin release reached 81% at 5 h. These releasing profiles indicate that these prepared CAANs could protect the majority of the amoxicillin against hydrolysis in gastric acid and that they exhibit a sustained amoxicillin release property in a pH-neutral environment. Additionally, the CAAN particle size dramatically increased with a net surface charge approaching zero at pH 7.0 (Figure 2b,c). Therefore, amoxicillin release from the CAANs may be due to the decrease in the ionic interaction between chitosan and amoxicillin and the collapse of the nanoparticle structure, which results in an aggregated structure and the large size formation of the drug carrier when the pH is higher than the pKa of chitosan. Moreover, the deprotonated alginate may simultaneously contribute to competing with amoxicillin for the binding sites of chitosan, resulting in more amoxicillin release at pH 7.0 (Figure 2d) [26].

### 3.3. In Vitro Mucoadhesion and Mucopenetration Evaluation

**Figure 3:**
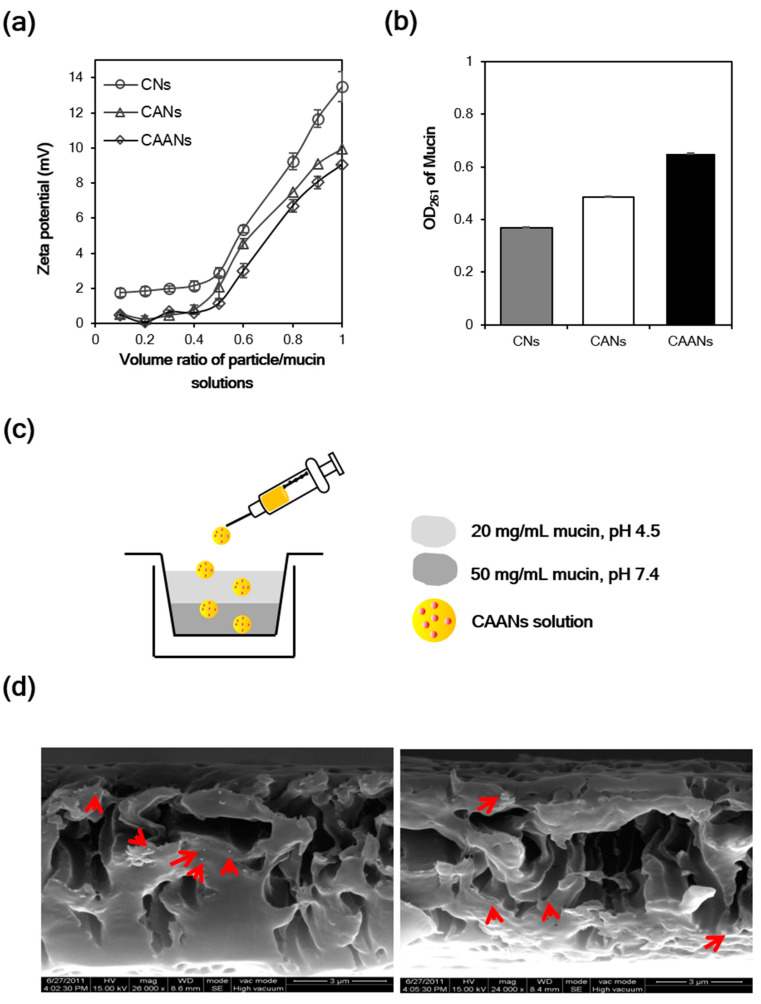
(**a**) Surface charge change of mucin particles in the CNs, CANs and CAANs solutions. (**b**) The OD value of unbound mucin at 261 nm after the contact with CNs, CANs and CAANs solutions. (**c**) Schematic illustration of the model used to mimic the mucus layer for the mucopenetration test. (**d**) Scanning electron microscope images of CAANs (red arrow) distributed in the mucin layer.

Some polymers such as chitosan, alginate, and carboxymethylcellulose possess mucoadhesive properties that can be used to lengthen the period of adhesion of the prepared drug carriers to the mucus layer [13]. The mucin particle method is a simple and rapid test for investigating whether the particles possess mucoadhesive properties by measuring the change in the zeta potential values of mucin particles upon the adsorption of particles [35]. Here, the surface charge of commercial mucin particles increased as the volume of particle solution increased, whether they interacted with CNs, CANs, or CAANs (Figure 3a). The zeta potential of the mucin particles that interacted with the CNs at a volume ratio of 1:1 was 13.5 mV, which is larger than that of the mucin particles that interacted with the CANs or CAANs (10.0 and 9.0 mV, respectively), revealing that the prepared CNs had best mucoadhesive properties. Although alginate is a mucoadhesive polymer, its mucoadhesive property is not as strong as that of chitosan (Figure S1). Therefore, this excellent mucoadhesive performance of the CNs, CANs, or CAANs may be contributed to from the presence of chitosan, which has excellent mucoadhesive properties due to a high content of amino groups and cationic charges and chain flexibility [13]. We also evaluated the mucoadhesive properties of the prepared nanoparticles by determining the unbound mucin in the supernatant after contact with mucin particles with CNs, CANs, or CAANs at 37 °C for 60 min [29]. The results shown in Figure 3b demonstrate that CNs possess the best mucoadhesive capacity, whereas CAANs have the lowest ability to bind mucin. This difference in the mucoadhesive capacity may be due to the high surface charge on CNs (26.3 mV at pH 2.5), which could result in increased particle–mucin complex formation to facilitate centrifugal separation.

The mucopenetration of CAANs through the mucus layer was evaluated using a mimic gastric mucosal system (Figure 3c). After their addition to the top of a mucin layer, the CAANs penetrated this mucin layer and were distributed, as shown by the SEM images (red arrow, Figure 3d). Although chitosan-based nanoparticles are easily trapped by the mucus layer, resulting in a limited particle penetration [36], the mucopenetration ability of CAANs could be improved by using a lower surface charge density particle, particles with a smoother surface, and smaller size to avoid ionic interactions and steric obstruction by mucin fibers [37].

### 3.4. In Vitro Bacterial Growth Suppression

**Figure 4:**
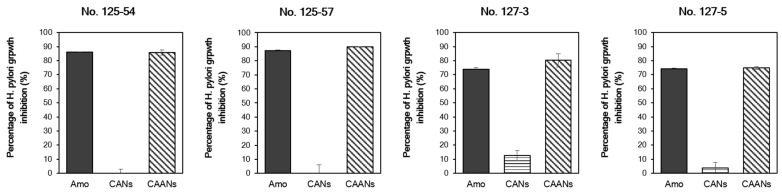
Percentage of *H. pylori* growth inhibition of the free amoxicillin, CAN, and CAAN treatments. The minimum inhibitory concentration (MIC) values of *H. pylori* strains No. 125-54, 125-57, 127-3, and 127-5 are ≥0.5, 0.25, 0.0625, and 0.0625 µg/mL, respectively. The treated concentration of amoxicillin was on the basis of the MIC value of each *H. pylori* strain.

We used four *H. pylori* strains with different MIC values to evaluate the in vitro anti-*H. pylori* activity of the prepared CANs and CAANs. The prepared CAANs inhibited *H. pylori* growth, as did free amoxicillin (Figure 4). Although the instantaneous release and activity of the encapsulated amoxicillin would be discouraged by the carrier structure, the hydrolysis of amoxicillin would be avoided, and the sustained release of amoxicillin could still be effective in the growth inhibition of *H. pylori*. Chitosan also has antibacterial activity [38]; therefore, the chitosan-based nanoparticles, such as the CANs, also inhibited the growth of *H. pylori*, particularly that of strains No. 127-3 and 127-5, with a low MIC value (≥0.0625 μg/mL).

### 3.5. In Vitro Cytotoxicity

**Figure 5:**
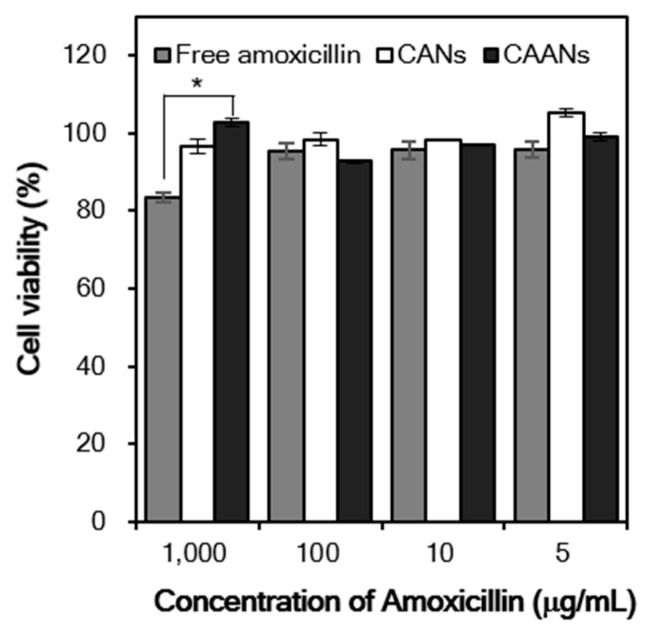
Relative cell viability of the NIH/3T3 fibroblasts treated with free amoxicillin, CANs, and CAANs at amoxicillin concentrations of 5, 10, 100, and 1000 µg/mL. *: *p* < 0.05.

In vitro cytotoxicity studies are usually the first step in determining the toxicity of drug carriers. Here, we evaluate the viability of NIH/3T3 fibroblasts after treatment with free amoxicillin, CANs, and CAANs at amoxicillin concentrations of 5, 10, 100, and 1000 μg/mL for 48 h. The viability of NIH/3T3 fibroblasts was always higher than 90%, except for the cells treated with 1000 μg/mL free amoxicillin (Figure 5). Amoxicillin acts as an antibiotic by inhibiting the cell wall formation of bacteria in order to stop bacterial growth [39]. Although mammalian cells do not have cell walls with a similar effect, amoxicillin may interact with DNA to induce DNA lesions and cellular toxicity [40,41]. These results indicated that this developed drug carrier could reduce the direct toxicity of high-dose amoxicillin to cells and, thus, would be a safe amoxicillin delivery system for oral administration.

### 3.6. Location of CAANs in the Gastrointestinal Tract

**Figure 6:**
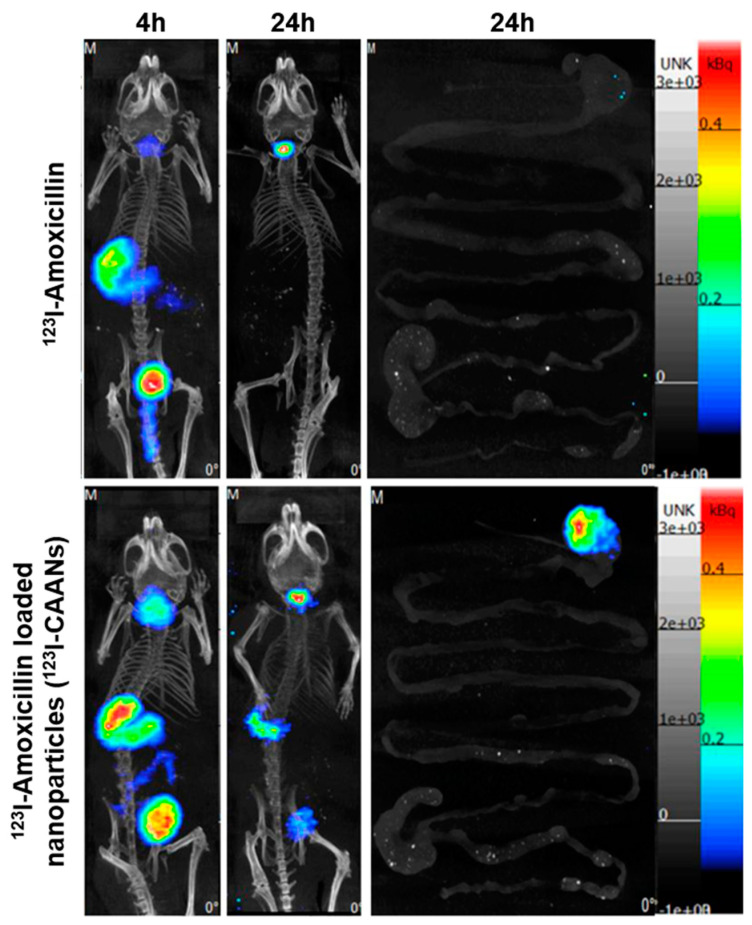
SPECT/CT images of mice at 4 and 24 h post oral administration of ^123^I-amoxicillin- and 123I-amoxicillin-loaded nanoparticles (^123^I-CAANs).

The staying time of amoxicillin in the stomach is short due to gastric motility; therefore, high doses of two or three antibiotics are frequently used to ameliorate the eradication rate of *H. pylori*, leading to increases in the incidence of side effects and decreases in therapeutic compliance [12]. To verify that the retention time of amoxicillin in the stomach could be extended by using chitosan-based nanoparticles, BALB/c mice were fed with ^123^I-amoxicillin and ^123^I-CAANs, and SPECT/CT images were obtained. Four hours after feeding the nanoparticles to the mice, large amounts of ^123^I-CAANs were found in the stomach (using a NanoSPECT/CT system) compared with the free ^123^I-amoxicillin-fed group (Figure 6). After 24 h, the high radioactivity intensity of ^123^I-amoxicillin was noticed in the stomach where ^123^I-CAANs were fed, but no radioactivity intensity could be observed in the stomach of mice fed with ^123^I-amoxicillin alone. These in vivo results indicate that the residence time of amoxicillin in the stomach can be prolonged using CAANs, and, consequently, high-dose and multiple antibiotics may not be required for *H. pylori* eradication.

### 3.7. Single Dose Pharmacokinetic Studies

**Figure 7:**
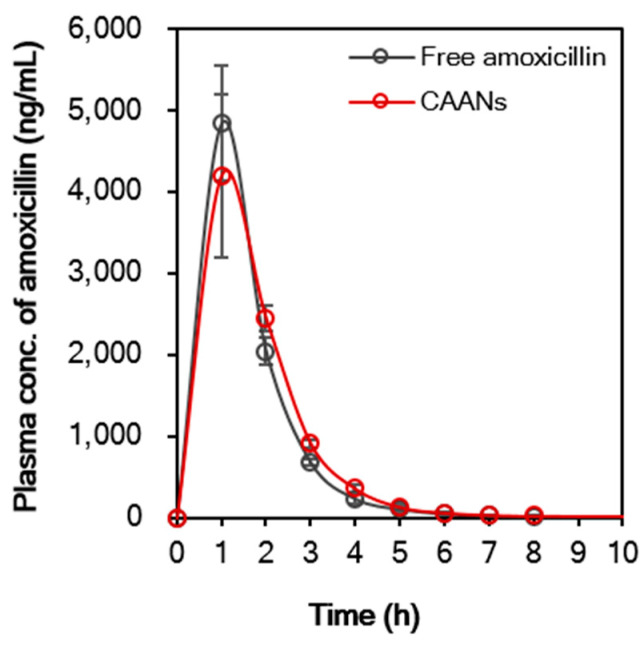
In vivo oral pharmacokinetic profile of free amoxicillin suspension and CAANs in a male CD^®^(SD)IGS rat model. All data points are expressed as mean ± SD (n = 4).

Encapsulation of amoxicillin in CAANs may affect the pharmacokinetic profile of the antibiotic. Figure 7 demonstrates that the pharmacokinetic profile of free amoxicillin differs slightly from that of CAANs. The maximum plasma concentration (C_max_) of amoxicillin in the free-amoxicillin-treated group was 4.8 mg/L, which was higher than that in the CAAN-treated group (4.2 mg/L) at 1 h post oral administration. After 2 h post oral administration, the plasma amoxicillin concentration of the CAAN-treated group remained above that of the free-amoxicillin-treated group. The pharmacokinetic parameters are listed in Table 1 and show that the mean residence time (MRT) for free amoxicillin and CAANs were 1.73 and 1.92 h, respectively, whereas the oral clearance (CL/F) for each treated group was 4.94 and 4.81 L/h/kg, respectively [42]. The volume of distribution of oral free amoxicillin was 7.28 L/kg and that of oral CAANs was 6.94 L/kg, indicating that the prepared CAANs should prevent the rapid spread of amoxicillin into body tissues and, thereby, reduce the risk of adverse effects [43]. Therefore, these pharmacokinetic results indicate that CAANs can increase the MRT of amoxicillin in the blood due to the protection of amoxicillin from hydrolysis in the stomach and enable sustained amoxicillin release for gastrointestinal absorption.

### 3.8. In Vivo Eradication Efficacy of H. pylori by CAANs

We used two *H. pylori* strains with different MIC values to evaluate the in vivo *H. pylori* eradication potency of the prepared CAANs. As shown in Table 2, the administration of CAANs for 7 days displayed 100% eradication efficacy on the low amoxicillin-resistant *H. pylori* (MIC value = 0.016 μg/mL), which was as high as that treated with standard triple therapy for 14 days according to the standard treatment protocol for *H. pylori* infection. Recently, the eradication rate of *H. pylori* has been significantly decreasing in the face of the rising prevalence of antibiotic resistance [4]. To determine the eradication efficiency of the CAANs on a highly antibiotic-resistant *H. pylori* strain, BALB/c mice were first infected with an *H. pylori* strain (NO. 125-54, with an MIC value of 0.5 μg/mL) that is highly resistant to amoxicillin and then used to evaluate the eradication efficacy of using CAANs. As shown in Table 3, the *H. pylori* eradication rate was 0% after the *H. pylori*-infected mice were treated with standard triple therapy for 14 days. Previous studies have demonstrated that the strategy for twice-daily treatment with amoxicillin does not achieve satisfactory treatment outcomes, suggesting that the standard triple therapy applied in this study could not supply an effective and stable amoxicillin plasma concentration against this highly amoxicillin-resistant *H. pylori* strain [11,44]. However, the feeding of CAANs to highly amoxicillin-resistant *H. pylori*-infected mice could improve the *H. pylori* eradication efficacy by up to 50%. It could be suggested that not only the designed CAANs could preserve amoxicillin from stomach acid damage, but also the residence time of CAANs in the stomach might be prolonged by the mucoadhesion property of chitosan. Additionally, the release of amoxicillin might be enhanced and sustained for direct activity against *H. pylori* by the competition of alginate for the chitosan cationic residues when the CAANs penetrate through the gastric mucus layer [26,45].

## 4. Conclusions

In this study, we have designed chitosan-based nanoparticles loaded with amoxicillin, a β-lactam antibiotic of the penicillin family (CAANs), as an *H. pylori* eradication platform. The incorporated alginate not only enhanced the retention time of CAANs in the stomach by utilizing their mucoadhesive properties but may also compete with amoxicillin for the cationic residues of chitosan to improve amoxicillin release when the prepared particles penetrate through the mucus layer. In vitro results indicate that the CAANs are biocompatible and retain the antibiotic activity of amoxicillin against *H. pylori* growth. The mucoadhesive property of chitosan and alginate enabled CAANs to adhere to the mucus layers and penetrate through the mucus to release amoxicillin in the space between the mucus layer and the gastric epithelium. In vivo results indicate that this designed nanoparticle could extend the amoxicillin’s residence time in the stomach and, thereby, result in an improved *H. pylori* eradication rate and a shorter treatment time. These CAANs, therefore, show potential for the effective treatment of highly antibiotic-resistant *H. pylori* infection using amoxicillin.

## Figures and Tables

**Table 1:** Pharmacokinetic parameters of free amoxicillin suspension and CAANs.

Parameters	Free Amoxicillin	CAANs
T_1/2_ (h)	1.02	0.996
C_max_ (mg/L)	4.8	4.2
T_max_ (h)	1	1
AUC_0-t_ (mg·h/L)	8.10	8.31
CL/F (L/kg/h)	4.94	4.81
V/F (L/kg)	7.28	6.94
MRT (h)	1.73	1.92

C_max_ = maximum plasma concentration, T_1/2_ = half-life, AUC_0-t_ = area under the curve, MRT = mean residence time, CL = clearance, V = volume of distribution, and F = bioavailability.

**Table 2:** In vivo eradication rate of different treatments of *H. pylori* with the MIC value of 0.016 µg/mL.

No.	1	2	3	4	5	6	7	8	9	10	Eradication Rate (%)
PBS	+	+	+	+	+	+	+	+	+	+	0
Standard triple therapy	−	−	−	−	−	−	−	−	−	−	100
CAANs	−	−	−	−	−	−	−	−	−	−	100

− indicates that no CagA-gene expression can be determined in the stomach tissue. + indicates that CagA-gene expression can be determined in the stomach tissue.

**Table 3:** In vivo eradication rate of different treatments of *H. pylori* with the MIC value of 0.5 µg/mL.

No.	1	2	3	4	5	6	7	8	9	10	Eradication Rate (%)
PBS	+	+	+	+	+	+	+	+	+	+	0
Standard triple therapy	+	+	+	+	+	+	+	+	+	+	0
CAANs	+	+	−	−	+	−	+	−	+	−	50

− indicates that no CagA-gene expression can be determined in the stomach tissue. + indicates that CagA-gene expression can be determined in the stomach tissue.

## Data Availability

The data presented in this study are available upon request from the corresponding author.

## Supplementary Materials

The following supporting information can be downloaded at: https://www.mdpi.com/article/10.3390/pharmaceutics14102117/s1, Figure S1: Surface charge change of mucin particles in the chitosan and alginate solutions.
