# Supplementary material for: Retention Time Extended by Nanoparticles Improves the Eradication of Highly Antibiotic-Resistant Helicobacter pylori
Source: Pharmaceutics. 2022 Oct 5;14(10):2117. doi: 10.3390/pharmaceutics14102117 (PMC9608011; doi:10.3390/pharmaceutics14102117)
Supplement: Supplementary file 1 [file pharmaceutics-14-02117-s001.zip › pharmaceutics-1909492-supplementary.pdf]

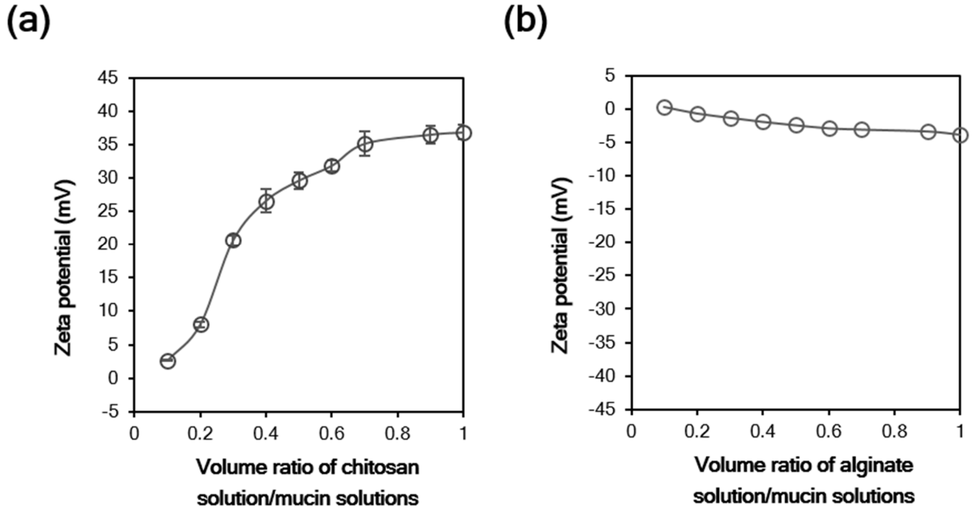

Figure S1. Surface charge change of mucin particles in the solutions of (**a**) chitosan and (**b**) alginate.
